# Factors influencing age at onset of colorectal polyps and benefit-finding after polypectomy

**DOI:** 10.1097/MD.0000000000035336

**Published:** 2023-09-29

**Authors:** Chen-Hong Feng, Qing Zhang, Juan Chen, Li-Qi Mao, Qian Sun, Ying He, Lin-Hua Yao

**Affiliations:** a Department of Gastroenterology, The First People’s Hospital of Huzhou, First Affiliated Hospital of Huzhou University, Huzhou, P.R. China; b Department of Gastroenterology, People’s Hospital of Wuxing District, Wuxing Branch of the First People’s Hospital of Huzhou, Huzhou, P.R. China; c Central Laboratory, The First People’s Hospital of Huzhou, First Affiliated Hospital of Huzhou University, Huzhou, P.R. China.

**Keywords:** benefit-finding, colonoscopy, colorectal polyps, polypectomy, risk factors

## Abstract

Screening, followed by colonoscopic polypectomy, has been widely performed in China. However, factors influencing age at onset of colorectal polyps and benefit-finding after polypectomy have been insufficiently studied or ignored. A total of 152 patients with colorectal polyps first detected in First Affiliated Hospital of Huzhou University from July to September 2022 were enrolled in this study. We selected 11 factors associated with the risk of colorectal polyps, including gender, body mass index, occupational stress, education level, income satisfaction, smoking, alcohol consumption, exercise frequency, diet, family history and polyp characteristics. Benefit-finding after polypectomy was obtained by follow-up for 142 of these patients. Multivariate linear regression analysis showed that being overweight (i.e., body mass index ≥25 kg/m^2^), higher education level, lower exercise frequency, and refrigerated food preference were associated with early-onset colorectal polyps. Patients with a preference for pickled food and age ≥50 years at first colorectal polyp detection had lower benefit findings after colonoscopic polypectomy. Colorectal polyps may develop earlier in people who are overweight, well-educated, exercise less, and prefer refrigerated food. In addition, patients who prefer pickled food and age at onset ≥50 years have lower benefit-finding requiring more attention in future colonoscopy follow-ups.

## 1. Introduction

Colorectal cancer (CRC) is the third most frequently diagnosed cancer and the most common cause of cancer-related death worldwide.^[1,2]^ From 1990 to 2019, the incidence and mortality of CRC are still rising in East Asia, including China.^[3,4]^ China has an immense CRC burden, accounting for about 30% of all new cases and all CRC-related deaths worldwide.^[5]^ Most CRC are considered to originate from preexisting adenomas. Hang D et al revealed that 60% to 80% of CRCs are formed through the traditional adenoma route.^[6]^ The initial small polyps could develop into cancer through genetic factors and long-term growth.^[7–9]^ Colorectal polyps may present with clinical symptoms such as blood in the stool and unformed stool, but most polyps may be asymptomatic.^[10]^ Therefore, colonoscopy screening as an effective intervention has been carried out in China and other countries with high incidence in the Asia-Pacific region.^[11,12]^ A population-based CRC screening study showed that nearly 1 in 5 subjects during the study period developed colorectal polyps.^[13]^ Previous studies have revealed that age, gender, race, smoking, obesity, alcohol, physical activity, nonsteroidal anti-inflammatory drugs, and dietary factors contribute to colorectal polyps, which involve society and lifestyle.^[13,14]^ Consequently, enhancing the identification of people at high risk for colorectal polyps can help prevent and manage CRC in advance.

Colonoscopic polypectomy is the mainstay of reducing the occurrence of adenoma-derived CRC. Endoscopic removal polyps offer many advantages of minimally invasive surgery, like less traumatic and fast recovery.^[15]^ However, the recurrence rate of polyps was as high as 20% to 50%.^[16,17]^ According to guidelines, post-polypectomy surveillance colonoscopy in 3 to 5 years is recommended in East Asia.^[18]^ The polyps continue to have a long-term impact and burden on patients after being removed. Moreover, the endoscopic operating environment, physicians, unfamiliar endoscopic equipment, and malignancy feedback are intense negative stressors that may lead to deleterious changes in psychological and physiological functioning, thus leading to patient depression and anxiety.^[19]^ Patients psychosomatic health and compliance with repeat colonoscopies may be compromised. Benefit-finding refers to positive changes in self-perception that may result from potentially harmful experiences.^[20]^ People with a history of colonoscopic polypectomy routinely evaluate their disease and lifestyle in everyday life. Such experiences are more likely to positively impact the meaning of the disease for these patients. Nevertheless, benefit findings with patients after colonoscopic polypectomy have not been studied.

This study focuses on factors related to the age at which colorectal polyps were first detected, intending to enhance the identification of people at high risk of colorectal polyps. Further follow-up analysis of benefit findings in patients undergoing colonoscopic polypectomy can help provide a basis for guiding patients to view the disease from a positive perspective and promote psychosomatic health.

## 2. Methods

### 2.1. Participants and indicator classification

**Figure 1.**
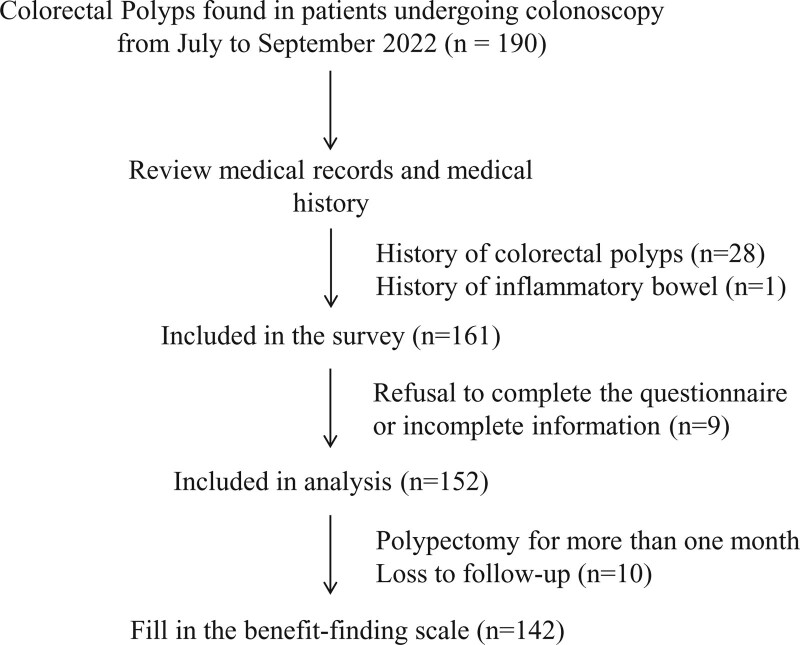
Flow chart of the study.

A total of 152 patients with colorectal polyps first detected in First Affiliated Hospital of Huzhou University from July to September 2022 were enrolled in this study. All patients underwent colonic polypectomy. Benefit-finding follow-up was performed 1 month after the polypectomy, with 142 cases finally collected (Fig. 1). All respondents volunteered to take the survey. The questionnaire was registered via a spreadsheet for statistical analysis only in a non-personalized. The ethics committee of First Affiliated Hospital of Huzhou University approved the study.

Eligible participants: patients with no history of colorectal polyps and inflammatory bowel disease by previous endoscopy; complete documentation of the location, number and size of colorectal polyps and pathological diagnosis after excision; patients who have no communication barriers and can answer investigation questions without the assistance of others; and the questionnaire information was filled out completely.

Body mass index (BMI) is one of the diagnostic bases for obesity according to WHO guidelines. A BMI of <25 is considered normal, and ≥25 is overweight. Dietary preferences of patients were specifically collected, including sweet, fried, spicy, refrigerated, and pickled food, all dichotomized as less frequent and more frequent. Specifically, those who ate more than twice a week were considered more frequent. We classified the location of polyps into the proximal colon, distal colon, and both. The size of the polyps included <1 cm and ≥1 cm groups, and the numbers of polyps ≥2 were considered multiple.

### 2.2. Benefit-finding

The benefit-finding scale (BFS) used in this study is a self-reported questionnaire comprising 19 items. This scale was developed by Tomich PL and Helgeson VS, modified and translated into Mandarin by Chinese scholars.^[21,22]^ Since Chinese speakers more easily understand localized expressions (e.g., “given my life better structure” changed to read, “make my life in good order”). Participants rated the extent of attitude and behavior changes caused by colorectal polyps, ranging from 1 (none at all) to 4 (very much). The BFS demonstrated acceptable reliability for the current sample (Cronbach alpha = 0.826).

### 2.3. Statistical analysis

All statistical analyses were performed using SPSS version 25.0. Categorical variables were expressed as frequencies and percentages and analyzed by Chi-square test. For numeric variables, means ± standard deviations were used to describe normally distributed data, while medians and interquartile range were used to describe skewed data. Comparisons were made using the *t* tests, analyses of variance, or Mann–Whitney *U* tests. In multiple linear regression, the variables suggested to be related to age at onset or sum BFS scores, with a *P* value of < .05, were entered. Correlations between variables were performed using Spearman rank correlation.

## 3. Results

A total of 152 cases were included, of which there were 94 (61.8%) males and 58 (38.2%) females (Table 1). The mean age was 55.11 ± 10.87 years, and the mean BMI was 23.47 ± 3.49 kg/m^2^. The subjects with colorectal polyps tend to have mild work stress, less than a high school education, average income or above, exercise ≥3 times per week, no drinking habits, and no family history. Regarding eating habits, the most favorite food is spicy, while most people eat fried food no more than twice a week.

**Table 1:** Characteristics of subjects with colorectal polyps (n = 152).

Variable	Number (%)	Age at onset	*t/F/Z/H*	*P*
Gender			−0.008	.994
Male	94 (61.8)	55.11 ± 10.92		
Female	58 (38.2)	55.12 ± 10.90		
Body mass index (kg/m^2^)			2.167	.032
<25	104 (68.4)	56.39 ± 10.57		
≥25	48 (31.6)	52.33 ± 11.11		
Work stress			5.357	.006
Mild	97 (63.8)	57.19 ± 10.39		
Moderate	27 (17.8)	52.37 ± 11.28		
Heavy	28 (18.4)	50.57 ± 10.50		
Educational level			6.151	.003
Less than high school	114 (75.0)	56.75 ± 9.89		
High school	23 (15.1)	51.78 ± 12.34		
University or above	15 (9.9)	47.80 ± 12.24		
Income satisfaction			0.248	.883
Satisfied	72 (47.37)	57.5 (63.75–48)		
Average	65 (42.76)	55 (60–51)		
Not Satisfied	15 (9.87)	55 (43–46)		
Exercise frequency			3.58	.008
Never or barely	34 (22.4)	50.32 ± 11.95		
1–3 times per mo	18 (11.8)	53.50 ± 13.38		
1–2 times per wk	14 (9.2)	52.14 ± 11.83		
3–5 times per wk	27 (17.8)	57.67 ± 9.87		
Daily	59 (38.8)	57.90 ± 8.46		
Smoking			3.594	.03
Never	75 (49.3)	54.89 ± 11.00		
Current	69 (45.4)	54.22 ± 10.75		
Former	8 (5.3)	64.88 ± 5.57		
Drinking			0.341	.711
Never	88 (57.9)	55.18 ± 11.62		
Current	58 (38.2)	54.66 ± 9.73		
Former	6 (3.9)	58.50 ± 11.11		
Diet			1.975	.05
Sweet food				
Eat less	99 (65.1)	56.37 ± 10.19		
Eat more	53 (34.9)	52.75 ± 11.78		
Fried food			3.636	<.001
Eat less	138 (90.8)	56.09 ± 10.32		
Eat more	14 (9.2)	45.43 ± 11.83		
Spicy food			3.033	.003
Eat less	89 (58.6)	57.30 ± 10.50		
Eat more	63 (41.4)	52.02 ± 10.71		
Refrigerated food			4.218	<.001
Eat less	116 (76.3)	57.08 ± 10.26		
Eat more	36 (23.7)	48.78 ± 10.49		
Pickled food			−0.931	.354
Eat less	107 (70.4)	54.58 ± 11.14		
Eat more	45 (29.6)	56.38 ± 10.21		
Family history			−2.49	.013
Yes	21 (13.82)	55 (65.5–48)		
No	131 (86.18)	56 (62–48)		

Table 1 presents the general characteristics of patients with colorectal polyps. BMI, work stress, education level, smoking status, frequency of exercise, fried food, spicy food, refrigerated food and family history were associated with the age at onset (Table 1). Further multiple linear regression analysis showed that BMI, education level, exercise frequency, and refrigerated food were independent risk factors for the presence of earlier-onset colorectal polyps (all *P* < .05, Table 2). Education level could affect health by influencing lifestyles. In Supplementary Table 1, http://links.lww.com/MD/J880, the correlation between education level and BMI, exercise, and refrigerated food was also analyzed. Lack of exercise was associated with higher education level (*P* = .036).

**Table 2:** Results of multifactorial linear analysis of patients age at first detection (n = 152).

Variable	Multivariate model				
	B	Std. Error	Standardized Coefficients	*t*	*P*
Constant	65.908	6.153	-	10.712	.000
Body mass index (kg/m^2^)	−4.164	1.682	−0.179	−2.476	.014
Work stress	−1.878	1.012	−0.136	−1.856	.065
Educational level	−3.432	1.193	−0.206	−2.878	.005
Exercise frequency	1.283	0.480	0.191	2.675	.008
Spicy food	−2.919	1.618	−0.133	−1.804	.073
Refrigerated food	−5.194	1.901	−0.204	−2.732	.007
Family history	4.523	2.336	0.144	1.936	.055

*F* = 9.004, *P* < .001, *R*^2^ = 0.304, Adjusted *R*^2^ = 0.271.

Table 3 displayed differences in polyp characteristics, including location, size, number, and pathological typing. There was a positive correlation between multiple polyps and age, with a median age of 55 years for solitary polyps and 58 years for multiple polyps, although it did not reach statistical significance (*P* = .051).

**Table 3:** Characteristics of colorectal polyps about the age at onset and gender.

	Age at onset			Gender	
	Age	*n* (%)	*P*	Male/female	*P*
Site			.751		.347
Proximal colon	54.86 ± 11.95	44 (28.9)		29/15	
Distal colon	54.64 ± 10.93	70 (46.1)		39/31	
Both	56.26 ± 9.59	38 (25.0)		26/12	
Size			.093		.237
<1 cm	54.17 ± 10.53	108 (71.1)		70/38	
≥1 cm	57.43 ± 11.47	44 (28.9)		24/20	
Number			.051		.232
Single polyp	55 (59–46)	59 (38.8)		33/26	
Multiple polyps	58 (65–50.5)	93 (61.2)		61/32	
Pathology			.140		.645
IN	56 (63.5–51.5)	77 (50.7)		49/28	
Non-IN	55 (62–44)	75 (49.3)		45/30	

IN, intraepithelial neoplasia.

Benefit-finding questionnaires were collected from 142 patients, of whom 86 (60.6%) were men and 56 (39.4%) were women. The mean age of the respondents was 54.75 ± 11.11 years, and the mean BFS was 46.15 ± 6.40. Items were listed in order of highest to lowest mean rating, showing descriptive information for all 19 questions in the BFS (Supplementary Table 2, http://links.lww.com/MD/J881). Total scores ranged from 34 to 70 (possible range of 19–76), with a mean total score of 46.15 (SD = 6.40). The mean scores of single items on the BFS scores ranged from 1.96 to 2.94, with an overall mean score of 2.43 (SD = 0.24). “Made my family closer and more united” was the item with the highest mean score (M = 2.94, SD = 0.52); “Make interested in participating in various activities” was the item with the lowest mean score (M = 1.96, SD = 0.91).

Supplementary Table 3, http://links.lww.com/MD/J882 showed the factors that influence the benefit finding after colonoscopic polypectomy. In the univariate models, 3 variables were correlated with the BFS scores: education level, pickled food, and age at onset. While in the multivariate models, preference for pickled food and age at onset ≥50 years were the independent risk factors for BFS scores (both *P* < .05, Table 4).

**Table 4:** Multiple linear regression analysis of the benefit-finding score (n = 142).

Variable	Multivariate model				
	*B*	Std. Error	Standardized Coefficients	*t*	*P*
Constant	51.775	2.929	-	17.676	.000
Educational level	1.579	0.848	0.158	1.862	.065
Preserved foods	−2.752	1.122	-0.198	−2.454	.015
Age at onset	−2.411	1.216	-0.170	−1.983	.049

*F* = 6.649, *P* < .001, *R*^2^ = 0.126, Adjusted *R*^2^ = 0.107.

## 4. Discussion

Most CRCs are sporadic and develop from precancerous lesions, that is, sporadic colonic adenomas/polyps.^[23]^ Any healthcare services for screening, removing, and preventing polyps are vital to prevent CRC. Our results showed that BMI ≥25 kg/m^2^, higher education level, lower exercise frequency, and refrigerated food preference were independently associated with age at onset of colorectal polyps, similar to other studies in East Asian populations.^[24–26]^ The median age of patients with a single and multiple polyp count was 55 and 58, respectively. Multiple polyps were detected more frequently in patients with older age, representing a higher potential risk of colorectal adenocarcinoma progression, although without reaching statistical significance (*P* = .051, Table 3).

Obesity is one of the important predisposing factors for many cancers and chronic diseases, representing a higher risk of progression.^[27]^ Only 7 people in this study had a BMI >30 kg/m^2^, so this group was not classified. This study demonstrated that BMI >25 was an independent risk factor for hyperplastic polyps, consistent with another research from China.^[25]^ Being overweight can increase the incidence of colorectal polyps and adenomas.^[24,28]^ A Meta-analysis covering 11 Asian individuals with a BMI of 25 to 30 kg/m^2^ and ≥30 kg/m^2^ had a significantly higher risk of colorectal adenoma than a BMI <25 kg/m^2^.^[29]^ These results suggest that the Chinese population may be more sensitive to the risk factors of weight gain in the pathogenesis of colorectal polyps. Interestingly, a study from Vietnam reported that being underweight rather than high weight, especially in male subjects, was associated with an increased risk of colorectal adenoma.^[30]^ One possible explanation was the low overall prevalence rate of obesity in Vietnam. Overall, the biological mechanism of overweight affecting the formation of colorectal adenoma was reticular, which may be influenced by many factors. Increased body fat can create a chronic subclinical inflammatory environment, altering the regional inflammatory state and increasing the abundance of specific microorganisms in the intestinal microbiota, thereby affecting colorectal adenoma formation.^[31–35]^ Metabolic factors have also been implicated in the development and progression of adenomatous polyps, involving increases in insulin-like growth factor 1 and insulin and the interaction of adiponectin and leptin.^[36,37]^ In summary, weight control through an active lifestyle may delay the age at onset of colorectal polyps.

Lack of physical activity, as one of the modifiable environmental risk factors, can induce CRC.^[38]^ Lieberman et al concluded that obesity alone was not a significant risk factor, while regular exercise can have a protective effect.^[39]^ Several studies have shown that increased physical activity reduces the risk of colorectal polyps, which was confirmed in the present study.^[40–42]^ Exercise probably achieved this goal through the mechanisms of energy balance and inflammation modulation.^[43]^ Prostaglandin E2 levels, proliferation and apoptosis of colonic epithelial cells are associated with energy balance.^[44]^ Inflammation regulation is related to inhibiting the cyclooxygenase-catalyzed synthesis of prostaglandins.^[45]^ Other involved mechanisms include enhancing immune function, reducing inflammation, reducing insulin levels and insulin resistance, decreasing intestinal transit time, and increasing vitamin D levels.^[46,47]^ In fact, public policymakers have been emphasizing the role of exercise as an integral part of a healthy lifestyle. Increasing the amount and frequency of sports might help delay the development of colorectal polyps.

Income, occupation, and education are the most common measures of socioeconomic status in epidemiological research.^[48]^ The present study evaluated the above 3 indicators and found that education level was negatively associated with age at onset of colorectal polyps, consistent with European populations.^[49]^ However, a study of North American had reported the opposite conclusion, potentially mediated by differences in populations.^[50]^ Interestingly, higher education level was significantly associated with physical inactivity in this study. Young or middle-aged and have the highest education level populations were the most inactive in China, contrary to developed countries.^[51,52]^ These educated adults were more likely to be engaged in sedentary jobs and lack the willingness to exercise after working long hours facing high stress. Higher educational backgrounds meant more motivation to undergo colonoscopy, which may also be a rational explanation for early onset.^[53]^

Unhealthy dietary habits can promote the development of colorectal neoplasia.^[54]^ We found that frequent consumption of refrigerated foods, including non-fresh foods, processed meats, and cold foods, may lead to an earlier age at onset of colorectal polyps. Long-term stored non-processed foods tend to have a higher content of nitrites and pathogenic bacteria.^[55]^ Nitrite reacts with amines, amides, and other nitrosative precursors in the gastrointestinal tract to form N-nitroso compounds, most as potent animal carcinogens associated with an increased risk of CRC.^[56–58]^ Nitrite/nitrate occurs naturally in plant foods and water as part of the nitrogen cycle and is also frequently used as an additive in processed meats.^[58]^ Fu Z et al concluded that processed meat was positively associated with all colorectal polyps.^[59]^ A large sample size prospective study in Japan showed that CRC was positively associated with processed meat consumption in males.^[60]^ A Meta-analysis revealed that processed red meat increases the risk of CRC more strongly than fresh red meat.^[61]^ The mechanisms involved may be related to fat, Heterocyclic Amines, Polycyclic Aromatic Hydrocarbons, Nitrite and N-Nitroso Compounds, Heme, fewer vegetables, and more calories.^[56,57]^ Reducing processed meat product intake is an effective way to prevent precancerous colorectal lesions. Changing the processing of these meat products may also help reduce the risk of early onset. Whether cold food affects colorectal polyps remains unclear and requires further study.

The concept of benefit finding stems from theories about the psychological response to threats and stress. It is broadly defined as a cognitive adaptation adopted by individuals to cope with threatening events such as cancer, manifested as less depression and more positive well-being by buffering negative emotions.^[62,63]^ The better benefit-finding ability helps to improve endocrine function and body immunity. On the other hand, it helps to guide healthy behavior and improve the perception of social support, thus promoting the rehabilitation of the disease.^[64]^ Recent benefit-finding studies have focused on various cancers and diseases with a long-term burden, such as rheumatoid arthritis and type 1 diabetes.^[65,66]^

Endoscopic screening is already widely performed in China, followed by removing polyps that can interrupt the progression of adenomas to CRC. Most people will experience transient abdominal distension, abdominal pain, nausea and vomiting after a colonoscopy.^[67,68]^ Many patients may experience preoperative anxiety and fear of pain and pathological malignancy.^[69]^ Some patients also suffer from intraoperative and postoperative complications of bleeding and perforation.^[70]^ Even after successful removal, patients may worry about the recurrence of the polyp, causing a long-term physical and mental burden. These psychological burdens may lead patients to escape follow-up and review, negatively impacting disease monitoring. According to our results, the benefit finding after polypectomy was lower in patients with colonic polyps who preferred pickled foods and were ≥50 years old at the time of onset.

Colonoscopy screening was recommended for average-risk individuals ≥50 years to reduce the incidence of advanced adenoma, CRC, and mortality from CRC.^[71]^ In the present study, this group represented a lower benefit finding. The more traumatic events impacted individuals previous abilities and identity (e.g., the feeling of immortality and expectations for the future, especially among young people), the more likely individuals were to develop an internal sense of growth after trauma.^[72]^ Young people have strong longevity expectations and feel more stressed and threatened after a traumatic experience. They are more willing to accept the learning and change of the process, which could help them better enjoy the present and prepare for life in the future. Older people tend to be more involved in managing other stressful life events, such as other comorbidities and bereavement pain, resulting in lower levels of benefit finding.^[63,73]^ Increased consumption of pickled foods has been demonstrated to enhance CRC risk.^[74–77]^ Moreover, we found that patients who ate pickled foods more than twice a week had lower benefits finding. In Chinese populations, the higher frequency of pickled foods intake increased the risks of mental stress.^[78]^ More clinical attention is needed to explore the positive psychology of this population from multiple perspectives. With the social system support, the benefit finding can be improved by guiding patients to focus on the positive aspects of the disease. There are long-term benefits to increasing motivation and adherence to colonoscopy review in people with a history of polypectomy.

There were several limitations of this study. First, this was a single-center study with a limited number of subjects included. Second, some of the statistical results may not be significant due to the sample size. Third, clinical characteristics were not assessed because most patients with colorectal polyps had no specific symptoms. Cognitive function and psychosocial function were also not assessed. Finally, interventions enhancing benefit-finding were not assessed, which remained to be further studied. Thus, future large follow-up studies with more indicators are needed to evaluate risk factors of colorectal polyps and benefit-finding after polypectomy.

## 5. Conclusions

BMI ≥25 kg/m^2^, higher education level, lower exercise frequency, and refrigerated food preference were risk factors for the earlier age of colorectal polyps. People with these characteristics may be at higher risk and require more aggressive screening strategies. In addition, patients with a preference for pickled foods and age of onset ≥50 years have a lower level of benefit finding and therefore require positive guidance. Future prospective cohort studies should validate these results and illuminate the underlying mechanisms.

## Author contributions

**Conceptualization:** Chen-Hong Feng, Qing Zhang, Juan Chen, Li-Qi Mao, Lin-Hua Yao.

**Data curation:** Chen-Hong Feng, Qing Zhang, Juan Chen, Li-Qi Mao.

**Formal analysis:** Chen-Hong Feng, Qing Zhang, Juan Chen, Li-Qi Mao, Lin-Hua Yao.

**Funding acquisition:** Lin-Hua Yao.

**Investigation:** Chen-Hong Feng, Qing Zhang, Juan Chen, Li-Qi Mao, Qian Sun, Ying He, Lin-Hua Yao.

**Methodology:** Li-Qi Mao, Qian Sun, Ying He, Lin-Hua Yao.

**Project administration:** Li-Qi Mao, Ying He, Lin-Hua Yao.

**Supervision:** Lin-Hua Yao.

**Visualization:** Chen-Hong Feng, Qing Zhang, Juan Chen, Li-Qi Mao.

**Writing – original draft:** Chen-Hong Feng, Qing Zhang, Juan Chen, Li-Qi Mao.

**Writing – review & editing:** Chen-Hong Feng, Qing Zhang, Juan Chen, Li-Qi Mao, Lin-Hua Yao.

## Supplementary Material






