# Supplementary material for: Factors influencing age at onset of colorectal polyps and benefit-finding after polypectomy
Source: Medicine (Baltimore). 2023 Sep 29;102(39):e35336. doi: 10.1097/MD.0000000000035336 (PMC10545222; doi:10.1097/MD.0000000000035336)
Supplement: Supplementary file 1 [file medi-102-e35336-s001.doc]

**Supplementary Table 1. Correlations between education level and other binary risk factors.**

| Factors | Spearman’s rank coefficient | *P* |
| --- | --- | --- |
| Body Mass Index | -0.102 | 0.213 |
| Exercise | -0.170 | 0.036 |
| Refrigerated food | 0.035 | 0.665 |
