# Supplementary material for: Factors influencing age at onset of colorectal polyps and benefit-finding after polypectomy
Source: Medicine (Baltimore). 2023 Sep 29;102(39):e35336. doi: 10.1097/MD.0000000000035336 (PMC10545222; doi:10.1097/MD.0000000000035336)
Supplement: Supplementary file 2 [file medi-102-e35336-s002.doc]

**Supplementary Table 2. Descriptive information for responses to benefit-finding scale.**

| Item | Description | M (SD) | Range |
| --- | --- | --- | --- |
| 11  10  6  12  5  8  2  9  14  16  13  19  1  17  15  4  3  7  18 | Brought my family close together  Made my life in good order  Made me more aware and concerned for the future of my family  Let me do things more efficiently  Made us more in charge of ourselves as a family  Made me a more responsible person  Made me more sensitive to family issues  Made me realize the importance of planning for my family’s future  Helped me to budget my time better  Taught me to be patient  Helped me take things as they come  Led me to cope better with stress and problems  Showed me that all people need to love and be loved  Taught me to control my temper  Made me more grateful for each day  Taught me that everyone has a purpose in life  Led me to be more accepting of things  Taught me how to adjust to things I cannot change  Renewed my interest in participating in different activities | 2.94 (0.52)  2.76 (0.58)  2.66 (0.75)  2.65 (0.65)  2.63 (0.71)  2.57 (0.68)  2.51 (0.71)  2.46 (0.70)  2.42 (0.61)  2.39 (0.71)  2.39 (0.68)  2.39 (0.63)  2.32 (0.57)  2.29 (0.74)  2.27 (0.68)  2.22 (0.70)  2.18 (0.70)  2.14 (0.65)  1.96 (0.91) | 1-4  1-4  1-4  1-4  1-4  1-4  1-4  1-4  1-4  1-4  1-4  1-4  1-4  1-4  1-4  1-4  1-4  1-4  1-4 |
