# Supplementary material for: Factors influencing age at onset of colorectal polyps and benefit-finding after polypectomy
Source: Medicine (Baltimore). 2023 Sep 29;102(39):e35336. doi: 10.1097/MD.0000000000035336 (PMC10545222; doi:10.1097/MD.0000000000035336)
Supplement: Supplementary file 3 [file medi-102-e35336-s003.doc]

**Supplementary Table 3. Univariate analysis of benefit-finding in patients with colorectal polyps (*n* = 142).**

|  | *n* (%) | Benefit-finding scale | | *Z* / *H* | *p* |
| --- | --- | --- | --- | --- | --- |
| **Gender** |  |  | -0.767 | | 0.443 |
| male | 86 (60.6) | 45 (49-41) |  | |  |
| female | 56 (39.4) | 45 (51-42) |  | |  |
| **Body mass index**  **(kg/m^2^)** |  |  | -0.575 | | 0.565 |
| < 25 | 95 (66.9) | 45 (52-41) |  | |  |
| ≥ 25 | 47 (33.1) | 44 (48-42) |  | |  |
| **Work stress** |  |  | 0.331 | | 0.565 |
| Mild | 89 (62.7) | 45 (49.5-42) |  | |  |
| Moderate | 25 (17.6) | 45 (50.5-41.5) |  | |  |
| Heavy | 28 (19.7) | 43.5 (49-41) |  | |  |
| **Educational level** |  |  | 8.940 | | 0.011 |
| Less than high school | 108 (76.1) | 44 (48-41) |  | |  |
| High school | 21 (14.8) | 47 (51.5-43.5) |  | |  |
| University or above | 13 (9.2) | 49 (54-45.5) |  | |  |
| **Income satisfaction** |  |  | -1.555 | | 0.120 |
| Satisfied  Average | 66 (46.5)  62 (43.7) | 46 (51-42.75)  44 (49-41) |  | |  |
| Not Satisfied | 14 (9.9) | 44 (49.25-40.75) |  | |  |
| **Exercise frequency** |  |  | 5.188 | | 0.269 |
| Never or barely | 34 (23.9) | 45.5 (48.5-43.75) |  | |  |
| 1-3 times a month | 17 (12.0) | 42 (45-40) |  | |  |
| 1-2 times per week | 13 (9.2) | 47 (50-40.5) |  | |  |
| 3-5 times per week | 26 (18.3) | 46 (50.25-40.75) |  | |  |
| Daily | 52 (36.6) | 44.5 (50.75-42) |  | |  |
| **Smoking** |  |  | 0.864 | | 0.649 |
| Never | 71(50.0) | 45 (51-42) |  | |  |
| Current | 64(45.1) | 44.5 (48-41) |  | |  |
| Former | 7(4.9) | 47 (52-42) |  | |  |
| **Drinking** |  |  | 0.158 | | 0.924 |
| Never | 81 (57.0) | 45 (51-42) |  | |  |
| Current | 55 (38.7) | 45 (51-42) |  | |  |
| Former | 6 (4.2) | 45.5 (47.75-40.75) |  | |  |
| **Diet** |  |  |  | |  |
| **Sweet food** |  |  | -1.312 | | 0.189 |
| Eat less | 91 (64.1) | 44 (49-41) |  | |  |
| Eat more | 51 (35.9) | 45 (49-44) |  | |  |
| **Fried food** |  |  | -0.841 | | 0.400 |
| Eat less | 130 (91.5) | 45 (49-41) |  | |  |
| Eat more | 12 (8.5) | 46 (49.75-42.5) |  | |  |
| **Spicy food** |  |  | -0.252 | | 0.801 |
| Eat less | 82 (57.7) | 45 (50-41.75) |  | |  |
| Eat more | 60 (42.3) | 44.5 (49-41.25) |  | |  |
| **Refrigerated food** |  |  | -0.889 | | 0.374 |
| Eat less | 107 (75.4) | 45 (49-41) |  | |  |
| Eat more | 35 (24.6) | 45 (49-43) |  | |  |
| **Pickled food** |  |  | -2.707 | | 0.007 |
| Eat less | 99 (69.7) | 46 (51-43) |  | |  |
| Eat more | 43 (30.3) | 43 (47-41) |  | |  |
| **Family history** |  |  | -1.528 | | 0.127 |
| Yes | 20 (14.1) | 46.5 (56-41.75) |  | |  |
| No  **Age at onset**  < 50  ≥ 50 | 122 (85.9)  40 (28.2)  102 (71.8) | 44.5 (49-41.75)  46 (53-43)  44.5 (48.25-41) | -2.067 | | 0.039 |
